# Contrastive learning for neural fingerprinting from limited neuroimaging data

**DOI:** 10.3389/fnume.2024.1332747

**Published:** 2024-11-13

**Authors:** Nikolas Kampel, Farah Abdellatif, N. Jon Shah, Irene Neuner, Jürgen Dammers

**Affiliations:** ^1^ Institute of Neuroscience and Medicine (INM-4), Forschungszentrum Jülich GmbH, Jülich, Germany; ^2^ Faculty of Medicine, RWTH Aachen University, Aachen, Germany; ^3^ Jülich Aachen Research Alliance (JARA) – CSD – Center for Simulation and Data Science, Aachen, Germany; ^4^ Faculty of Mathematics, Computer Science and Natural Sciences, RWTH Aachen University, Aachen, Germany; ^5^ Jülich Aachen Research Alliance (JARA) – BRAIN – Translational Medicine, Aachen, Germany; ^6^ Institute of Neuroscience and Medicine (INM-11), Jülich Aachen Research Alliance (JARA), Forschungszentrum Jülich GmbH, Jülich, Germany; ^7^ Department of Neurology, University Hospital RWTH Aachen, Aachen, Germany; ^8^ Department of Psychiatry, Psychotherapy and Psychosomatics, RWTH Aachen University, Aachen, Germany

**Keywords:** deep learning, contrastive learning, neural fingerprinting, fMRI, resting-state, functional connectivity, limited data

## Abstract

**Introduction:**

Neural fingerprinting is a technique used to identify individuals based on their unique brain activity patterns. While deep learning techniques have been demonstrated to outperform traditional correlation-based methods, they often require retraining to accommodate new subjects. Furthermore, the limited availability of samples in neuroscience research can impede the quick adoption of deep learning methods, presenting a challenge for their broader application in neural fingerprinting.

**Methods:**

This study addresses these challenges by using contrastive learning to eliminate the need for retraining with new subjects and developing a data augmentation methodology to enhance model robustness in limited sample size conditions. We utilized the LEMON dataset, comprising 3 Tesla MRI and resting-state fMRI scans from 138 subjects, to compute functional connectivity as a baseline for fingerprinting performance based on correlation metrics. We adapted a recent deep learning model by incorporating data augmentation with short random temporal segments for training and reformulated the fingerprinting task as a contrastive problem, comparing the efficacy of contrastive triplet loss against conventional cross-entropy loss.

**Results:**

The results of this study confirm that deep learning methods can significantly improve fingerprinting performance over correlation-based methods, achieving an accuracy of about 98% in identifying a single subject out of 138 subjects utilizing 39 different functional connectivity profiles.

**Discussion:**

The contrastive method showed added value in the “leave subject out” scenario, demonstrating flexibility comparable to correlation-based methods and robustness across different data sizes. These findings suggest that contrastive learning and data augmentation offer a scalable solution for neural fingerprinting, even with limited sample sizes.

## 1 Introduction

Neural fingerprinting is a technique that enables the identification of individuals based on their unique brain activity patterns, affording detailed insight into how the brain functions and processes information and offering applications in personalized medicine.

Neuroscientific research has demonstrated promise in the identification of individuals through the inference of their neural fingerprints using a number of imaging methodologies. Notably (1), conducted magnetoencephalography (MEG) fingerprinting using functional and structural connectivity features to successfully obtain neural fingerprints, while (2) explored the impact of various frequency bands and brain connections on MEG functional connectivity (FC) fingerprints, comparing it to functional magnetic resonance imaging (fMRI) data from the same subjects. Both studies employed Pearso's correlation coefficient as an identifiability measure.

A comprehensive review published by (3) outlined techniques utilized for extracting neural fingerprints from neuroimaging data. Although electroencephalography (EEG) was the earliest modality employed for fingerprinting (2001), MRI-based fingerprinting studies (starting from 2015) accounted for 38% of the surveyed published works, suggesting its substantial relevance in the field.

In the context of functional neuroimaging, FC is a commonly used feature as it provides information about the communication between brain regions (4). There are several methods for deriving the FC profiles, depending on the neuroimaging modality. Many advancements in fMRI FC fingerprinting were made possible through the data provided by the Human Connectome Project (HCP) (5). For example, based on rs-fMRI data from the HCP (6), used a correlation coefficient as a measure of identifiability by comparing a target FC profile against a sample database of FC profiles. Amico and Goñi (7) aimed to improve on the results of this work by reconstructing the functional connectivity profiles using a principal component analysis (PCA) decomposition algorithm to extract different components associated with the whole population and then using the correlation coefficient as the identifiability measure. A more recent work, which uses the same HCP data (8), looked into the optimal time scale for identification by calculating dynamic FC and using edgewise inter-class correlation (ICC) as a statistical measure to identify individuals. Li et al. (9) adapted the measure of identifiability used by Finn et al. (6) and examined the impact of sample size and atlas granularity on fingerprinting accuracy by presenting a framework for feature selection designed to extract higher-quality subject-specific FC information for neural fingerprinting from rs-fMRI.

All of the aforementioned studies utilized correlation matrices as measures of identification and demonstrated the high accuracy and robustness of neural fingerprinting on rs-fMRI functional connectivity. Additionally, Sarar et al. (10) employed shallow feedforward neural networks on functional connectivity derived from 20-s rs-fMRI segments, achieving an accuracy of ≥99.5% across two sets of 100 subjects using 379 regions of interest (ROIs). This result demonstrates the potential of deep learning for precise subject identification based on short data segments.

Recent studies have progressed beyond static FC profiles to capture the temporal dynamics in neuroimaging data. For instance, Kampel et al. (11) applied a machine learning algorithm specifically designed for multivariate time series data for MEG fingerprinting, enabling for the extraction of temporal features that enhance identifiability. Similarly, Wang et al. (12) developed a Convolutional Recurrent Neural Network (ConvRNN) to capture spatio-temporal information from rs-fMRI, further improving individual identification by modeling dynamic functional connectivity.

Although deep learning is of great interest in neuroimaging research due to its unexplored potential, there are many challenges preventing rapid progress, most notably high dimensionality (i.e., spatial and temporal dimensions) and low sample size (13). Additionally, it is a common concern that deep learning models trained on neuroimaging data suffer from overfitting (14). Although the acquisition of a large neuroimaging database could serve as a remedy for most of the challenges, such databases are not widely accessible. However, one could artificially augment the size of the training data by means of slicing long recordings into several smaller ones. Alternatively, one could employ a deep learning training paradigm that is better suited for cases where the number of target classification categories is large compared to the available training samples per category. Namely, contrastive learning has shown great promise as a discriminative learning approach when the model is trained to identify similarities between representations of input pairs that are contrasted against each other. The work of Hassanzadeh et al. (15) explored the spatial variability of individuals by conducting a fingerprinting study using fMRI independent component analysis (ICA)-based spatial maps. They employed a 3-dimensional convolutional neural network (CNN) as the embedding network in a siamese neural network framework (16) with a contrastive loss function. While they had access to a large database of 12,000 subjects, the contrastive learning paradigm was convenient for conducting a pairwise comparison study on spatial network maps for neural fingerprinting. Similarly, to our study, Wang et al. (17) used fMRI FC in a contrastive deep learning paradigm. However, in this study, data from 596 patients were used and a graph neural network (GNN) was applied for population-based fMRI classification. The contrastive paradigm was utilized in this context to simply explore its performance for a population-based FC classification with graph networks.

The objective of our study is to enhance model-based neural fingerprinting by overcoming two significant challenges: the necessity for effective model retraining and the constraints of small datasets. To address the issue of model retraining, we employed a leave-subject-out approach, which was made possible by contrastive triplet loss. To offset the impact of limited data, we incorporated various levels of data augmentation. We evaluated three distinct model configurations: conventional cross-entropy loss, contrastive triplet loss, and a leave-subject-out configuration. For each, we compared it against a baseline correlation method. Furthermore, we applied different levels of data augmentation to all configurations to comprehensively assess their robustness and performance.

## 2 Methods

### 2.1 Dataset

The open-source Mind-Brain-Body Dataset – LEMON from the Max Planck Institute of Leipzig, Germany (18) was used for data analysis. The original purpose of the 2019 study was to investigate the association between psychological factors and somatic health by acquiring physiological and psychological assessments, as well as neuroimaging data, to produce the “Leipzig Study for Mind-Body-Emotion Interactions” (LEMON) dataset. This LEMON was collected between 2013 and 2015 and included 227 individuals in total, divided into two groups: young adults and older adults. The young group had an age range of 20–35 (*N* = 153, mean age 25.1 ± 3.1, 45 females), and the older group had an age range of 59–77 (*N* = 74, mean age 67.6 ± 4.7, 37 females). All participants took part in a pre-screening interview, a two-day assessment to collect data, and a follow-up (18).

The MRI scanning was carried out on a 3 Tesla scanner (MAGNETOM Verio, Siemens Healthcare GmbH, Erlangen, Germany). The imaging protocol lasted for 70 min and included rs-fMRI, quantitative T1 (MP2RAGE), T2-weighted imaging, FLAIR, SWI/QSM, and DWI. The rs-fMRI scans were acquired for a total of 15 min, and the subjects were instructed to remain at rest with their eyes open. In our study, we selected all subjects from the LEMON dataset from which four resting-state recordings, a T1-weighted image, and a low-resolution FLAIR image was available. This resulted in a subset of 138 subjects.

### 2.2 Preprocessing

To prepare the rs-fMRI data for analysis, the following series of preprocessing steps were performed by the owners of the LEMON dataset according to the pipeline detailed in (19): (i) Motion Correction, (ii) Distortion Correction, (iii) Coregistration, (iv) Combined Transformations, (v) Masking, (vi) Nuisance Regression, (vii) Physiological Noise Removal, (viii) Temporal Filtering and Normalization, and (ix) Standard Space Projection. The preprocessed data was made accessible in both the subjects’ original structural space and in Montreal Neurological Institute (MNI) standard space, alongside the brain mask of the subject and all pertinent regressors employed for denoising. Following preprocessing, the data resolution was 2 mm in MNI 152 space, with each run of rs- fMRI lasting around 15 min.

### 2.3 Functional connectivity

The pivotal step in the analysis involves computing functional connectivity matrices, also referred to as connectomes. These connectomes were derived from the time series data and represent the temporal dynamics of the neural activity across specific ROIs. These ROIs were delineated based on the Multi-Subject Dictionary Learning (MSDL) atlas, which comprehensively maps brain regions to investigate brain spontaneous activity (20). Utilizing this atlas, 39 nodes across 17 resting-state networks were identified. For each subject, the multivariate time series data were bandpass filtered from (0.01–0.1) Hz and subsequently normalized using z-scores. The resulting preprocessed time series were then used to (i) generate functional connectivity matrices through Pearson correlation, capturing the pairwise correlations among the selected ROIs and serving as our baseline calculation, and (ii) utilize the temporal information of each ROI for subsequent deep learning-based analysis. To facilitate the implementation process, the Pearson correlation was calculated as a deterministic operation within the network. This approach enables dynamic adjustments to varying time series durations and allows for efficient on-the-fly data augmentation without the need to precompute connectivity matrices.

### 2.4 Data splitting

The evaluation of the deep learning models (DLM) investigated in this study primarily employed two different partitioning scenarios that we refer to as Leave Run Out (LRO) and Leave Subject Out (LSO). As observed in related work (10, 12), LRO-based splitting is commonly used in neural fingerprinting tasks. When applying LRO partitioning, the data set is split based on runs or sessions per subject; certain runs for subjects are designated for training, and the remaining runs for the same subjects are designated for validation. This approach emphasizes the ability to generalize on within-subject variations and is, therefore, practical for assessing temporal changes or session-based variability in the imaging data.

Compared to the widespread use of LRO-splitting in neural fingerprinting tasks, the adoption of LSO as an alternative data-splitting method has been notably less common, except in the work of Hassanzadeh et al. (15). In LSO-splits, the dataset is divided in a way that all runs associated with a subgroup of subjects are utilized for training the model, while the remaining data from unseen subjects are set aside for validation. This method is advantageous because excluding clusters of related observations helps minimize bias and improves the generalizability of the model across various individuals (21).

### 2.5 Baseline: correlation analysis

To establish a baseline, i.e., a reference, for the deep learning-based neural fingerprinting, we adopted the classification procedure described in (6) and used comparisons between FC vectors derived from all four resting-state sessions. Each comparison involved a single session, which was identified as the query, and one or more target sessions. Each FC vector from the query session was then evaluated against all other vectors from the target sessions. A Pearso's correlation was used to quantify the similarity between FC vector pairs. Correct subject identification was deemed to be achieved when the query vector had the highest absolute correlation coefficient to one of its corresponding target vectors. A correct classification was given a score of 1, while an incorrect classification was given a score of 0. The average accuracy was calculated by taking the average of the scores across all subjects and across all possible comparisons. In the following, we will refer to this type of classification procedure as the “baseline” method for comparison, as it is widely used in neural fingerprinting classification scenarios (6, 9).

### 2.6 Deep learning-based classification

**Figure 1:**
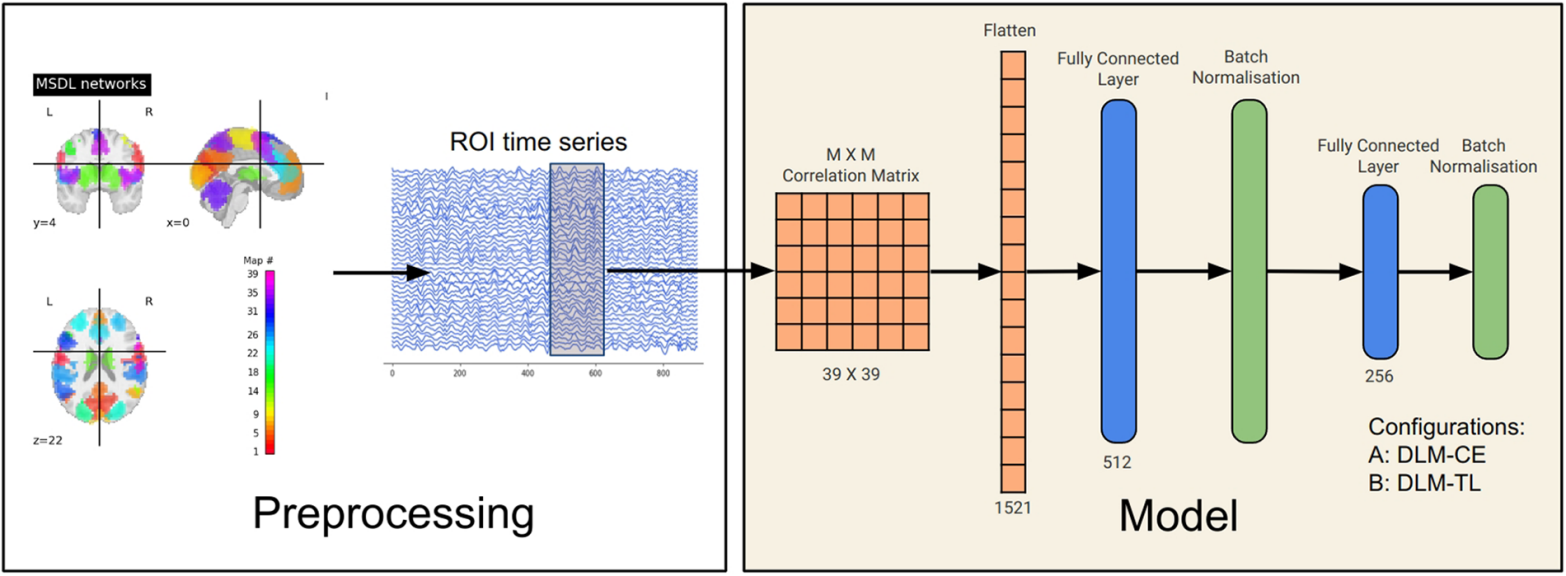
Schematic representation of the processing and model pipeline for neural fingerprinting. Starting with MSDL-defined brain networks, regional brain activation time courses are extracted. A frame overlaid on these courses indicates data augmentation. The time courses are then transformed into a connectivity matrix, which is then fed into two fully connected layers with subsequent batch normalization. The output layer is then passed through a Softmax activation layer or an L2-normalization layer, depending on the type of model configuration used, i.e., LRO or LSO-based classification (see Sections 2.6.1 and 2.6.2).

The architecture of the DLMs employed in this study comprises two primary components: the backbone and the head of the model. The backbone serves as the foundational part of the model structure and is responsible for feature extraction (Figure 1). It is a feed-forward neural network with its first hidden layer set to 512 units, configured based on guidelines and architectures established in previous research (10). To better suit the specific requirements of our study, an additional second hidden layer with a size of 256 units was added, both accompanied by a batch normalization layer (Figure 1).

#### 2.6.1 DLM with cross-entropy loss

The first variant of the model, referred to as “Deep Learning Method with Cross-Entropy Loss” (DLM-CE), integrates neural fingerprinting as a multi-class classification task. The binary cross-entropy loss was used as the objective function and was coupled with a softmax activation function. All subjects were represented by a corresponding one-hot coded label. An output layer equal to the number of subjects to be identified was added at the end of a backbone architecture (Figure 1). Training was performed with a batch size of 276.

#### 2.6.2 DLM with contrastive triplet loss

The second DLM variant utilizes a triplet loss function (22) for classification, with the goal of enhancing the adaptability of the classification model in scenarios where the identification of subjects not encountered during training is facilitated. Here, the encoder network replicates the backbone architecture of the DLM-CE, but with L2 normalization applied to the final layer instead of attaching a class coding layer (Table 1). The DLM with triplet loss (DLM-TL) is based on contrastive learning and was designed to calculate a distance metric between any instances of the input data. The methodology consists of two distinct steps. First, embeddings are generated using an encoder network. Then, these embeddings are utilized for classification based on vector similarity, specifically the distances between embeddings of FC. This set of embeddings includes an anchor subject, denoted a $a_{i}$, a positive instance with FC from the same subject but from a different run $p_{i}$, and a negative instance with FC from a different subject $n_{i}$. According to Schroff et al. (22), the formula for the triplet loss is given as shown in Equation 1:

(1) $$\|f(x_{i}^{a})-f(x_{i}^{p}){\|}_{2}^{2}+\alpha <\|f(x_{i}^{a})-f(x_{i}^{n}){\|}_{2}^{2}\;$$

**Table 1:** Model components and settings employed in the deep learning-based models used for neural fingerprinting.

Generalization	Model type	Size of last layer	Conditioning	Loss function
LRO	DLM-CE	138	Softmax activation	Cross entropy loss
LRO, LSO	DLM-TL	256	L2 normalisation	Triplet loss

Here, *α* represents a margin enforced between positive and negative pairs, while f denotes the embedding function. The overall loss, L, to be minimized is expressed as shown in Equation 2:

(2) $$L=\sum_{i}^{N}\;[\|\;f(x_{i}^{a})-f(x_{i}^{p}){\|}_{2}^{2}-\|f(x_{i}^{a})-f(x_{i}^{n}){\|}_{2}^{2}+\alpha {]}_{+}$$

with *N* representing the total number of triplets in the training set. Essentially, the model attempts to minimize the distance between the anchor and the positive examples while maximizing the distance between the anchor and the negative examples (22). A semi-hard triplet mining strategy was used in conjunction with the ADAM optimizer to train the model (23).

In order to ensure accurate classification using triplet loss, it is necessary for the model to have access to at least two recordings per subject. The model generates embeddings for these recordings, and classification is considered successful when the distance between the anchor embedding and the positive embedding is smaller than the distance between the anchor embedding and any other negative embeddings in the set. This approach allows for the determination of accuracy not only for subjects seen during training (LRO) but also for subjects not seen during training (LSO), as long as there are at least two recordings available per subject in both the training and test sets.

### 2.7 UMAP visualization

To visualize the high-dimensional embeddings derived from our LSO_DML_TL model, we employed Uniform Manifold Approximation and Projection (UMAP) (24). The rationale behind using UMAP was to project the 256-dimensional embedding vectors into a two-dimensional space, facilitating the examination of the arrangement of subjects in the embedding space and the evaluation of clustering improvements for both training and unseen test subjects. Unlike demographic-based clustering approaches (e.g., grouping by age or gender), our analysis focuses on clustering recordings within the same individual. This approach enables us to assess the consistency and stability of neural fingerprints across multiple sessions. The UMAP configuration was established with the default parameters of the umap-learn 0.5.3 package (*n*_neighbors = 15, *n*_components = 2, metric = “euclidean”, and learning_rate = 1) to ensure an optimal balance between local and global structure preservation in the embeddings (24).

### 2.8 Cross-validation

Given the limited data set, it was deemed impractical to divide the data into the conventional three splits: training, validation, and test sets.

Instead, the following cross-validation schemes were used unless otherwise noted: For the DLM-CE method with LRO splitting (LRO_DLM-CE), models were trained on data from two out of the four available resting-state sessions and then evaluated on the remaining two. This process was performed iteratively for all six possible combinations of training and test sets. Accuracy was used as the primary performance measure. Each combination was repeated 10 times resulting in 60 calculations to account for variability introduced by factors such as data augmentation and initial weight settings.

To enhance the assessment of the LSO scenario in the DLM-TL method (LSO_DLM-TL), additional evaluation metrics were included. Specifically, in addition to accuracy, precision, recall, and F1 score were also calculated to provide a more comprehensive understanding of the mode's performance. Each possible evaluation was also repeated 10 times.

### 2.9 Data augmentation

To mitigate the risk of over-fitting, we used a data augmentation scheme specifically designed to introduce variability into the training data set, thereby improving the mode's ability to generalize to unseen data. In this approach, each training batch consisted of short, randomly selected time segments extracted from the entire time series data. For example, during each training run, a 3-min segment was randomly chosen from the entire 15-min resting state run. This random selection and subsequent computation of functional connectivity were done in real-time. This method enables the model to analyze distinct, smaller portions of the data in each training batch.

In order to determine the optimal duration of these time segments, we conducted a series of experiments. We varied the segment duration in increments of 90 s, and subsequently evaluated the performance of all model configurations using the metrics described in the previous section. To further measure the variability introduced by this random selection process, each training run was repeated ten times, allowing us to measure variations in the classification accuracy.

Moreover, a permutation *t*-test (25), as implemented in (26), was utilized to ascertain whether the methods exhibited significant differences. Both LRO_DLM_TL and LSO_DLM_TL were compared against LRO_DLM-CE, with the significance of each augmentation step being evaluated separately. To ensure robust statistical analysis, 100,000 repetitions were employed.

### 2.10 Split ratios

In a low sample size data regime, the ratio of data for the training and validation sets need to be chosen carefully. In this study, we examined the impact of the split ratio on performance by varying the proportion of subjects in the training and validation sets. Previous studies suggest that a smaller validation set may improve accuracy (9). To test this dependency within an LSO splitting, the LSO_DLM-TL classification performance was evaluated over 19 different training and validation set ratios, ranging from 5% to 95% with respect to the total number of subjects. Each proportion was tested ten times to control for variables such as subject randomization, weight initialization, and data augmentation. For data augmentation, the segment size was set to 270 s, as this parameter yielded optimal results (cf. Section 3.1). For comparison, accuracies were also calculated using correlation-based methods on the same data.

### 2.11 Sensitivity analysis for dataset size

To examine the influence of the sample size on the performance, we employed a fixed ratio of 2/3 for training to validation sets using the LSO_DLM-TL method. The evaluation began with 92 subjects for the training set and 46 subjects for the validation set. These numbers were then reduced in 10% increments until a minimum of eight subjects for the training set and four subjects for the validation set was reached. Each sample size condition was replicated 10 times in order to calculate the mean and standard deviation of model performance. Similarly to the previous section, the segment size for data augmentation was set to 270 s to optimize performance. In accordance with Section 2.10 accuracies were also computed using the baseline methods on the same subsets of data.

## 3 Results

### 3.1 Impact on the size of data augmentation

**Figure 2:**
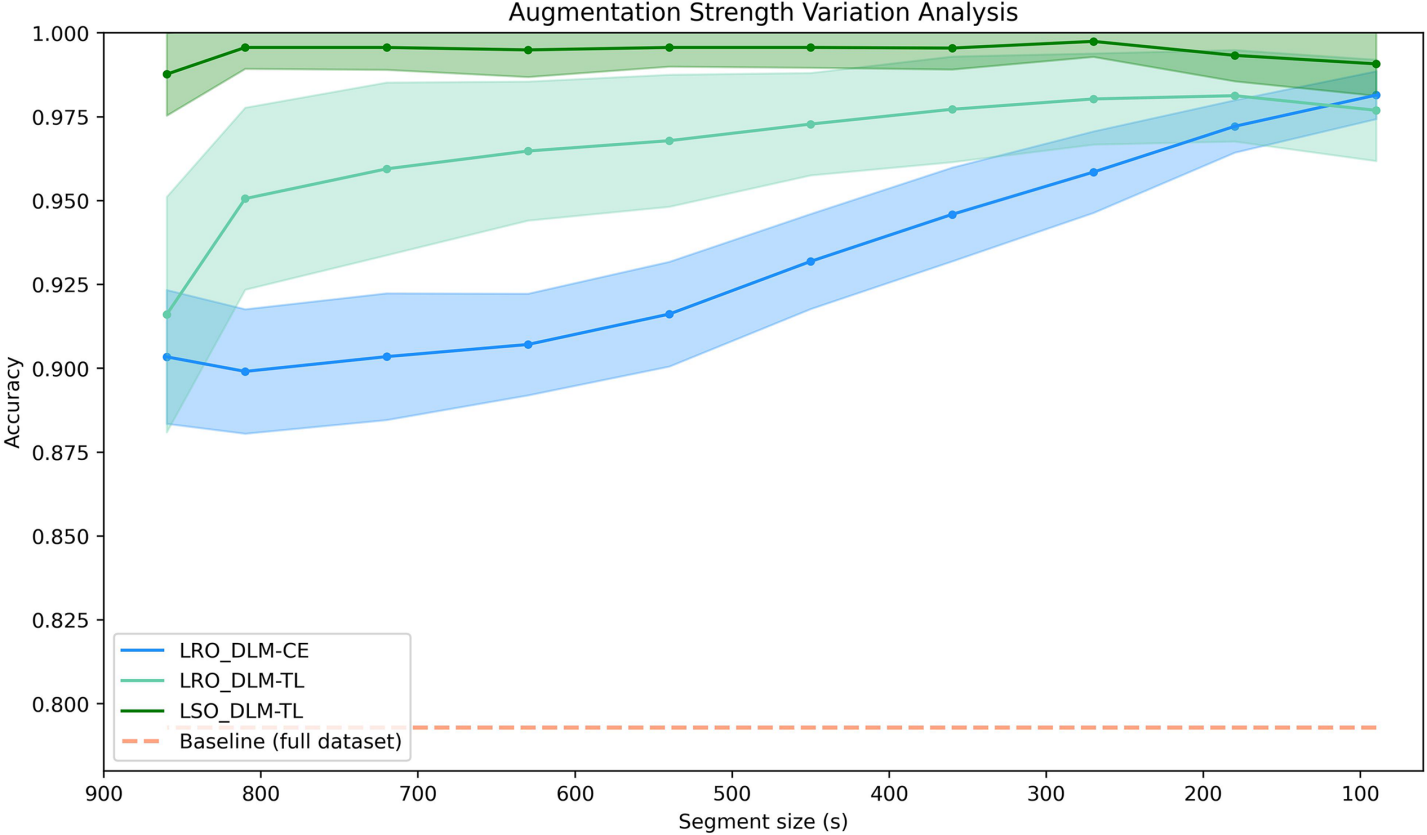
Classification performance depending on the length of the ROI time course. The graph illustrates the relationship between different segment sizes (in seconds) and the achieved classification accuracy for the LRO_DLM-CE model (blue line), LRO_DLM-TL model (green line), and LSO_DLM-TL model (light green line), with their respective standard deviations from 60 repetitions shaded accordingly. The orange dashed line represents the baseline accuracy utilizing correlation to compute the accuracy. Note that the *x*-axis is inverted, as smaller segment sizes corresponding to higher levels of data augmentation by using shorter but many more time segments.

Our analysis, utilizing various data lengths for data augmentation, shows a significant impact on model performance across all deep learning-based methods. Increasing the intensity of augmentation by reducing the size of the time segment led to notable improvements in identification accuracy and its variance. Figure 2 demonstrates that as data segment sizes decrease, model performance enhances, emphasizing its sensitivity to the duration and variability of the input data.

The classification results shown in Figure 2 indicate that all deep learning models benefit from the random selection of shorter time segments, leading to a consistent increase in accuracy. The models achieve the highest performance under maximum augmentation, which involves selecting a larger number of time segments of 90 s duration randomly.

In contrast to the classical cross-entropy loss method (LRO_DLM-CE), both triplet loss-based models (LRO_DLM-TL and LSO_DLM-TL) showed improvements even for larger time segments with fewer data augmentation steps. Particularly, the LSO_DLM-TL method exhibited the best classification results, maintaining high accuracy across all levels of data augmentation. The classification results of the LSO_DLM-TL method also demonstrated to have the lowest variance across 60 repetitions among the methods and is the only one among the three that offers generalization capabilities.

The results of the applied permutation *t*-test revealed that the utilization of the triplet loss function leads to significant improvements (*p* < 0.01) across nearly all augmentation levels compared to the LRO_DLM-CE method. The only exceptions were observed in the shortest (90 s) and longest (875 s) time segments, with *p*-values of 0.099 and 0.753, respectively.

### 3.2 Performance benchmarking for LRO splitting

We investigated various LRO-split scenarios by examining the performance under two edge cases: minimum and maximum augmentation. The baseline accuracy in identifying subjects based on FC was found to be 79.3%. In contrast, the deep learning model utilizing cross-entropy loss (LRO_DLM-CE) demonstrated an average identification accuracy of 90.3% ± 1.8%, representing an improvement of approximately 11% over the baseline. The variant of the deep learning model using triplet loss (LRO_DLM-TL) also achieved high accuracies, with a mean accuracy of 91.7% ± 3.6%.

When the input data was augmented using a random time segment of 90 s, both models demonstrated improved performance. Specifically, the LRO_DLM-CE model achieved an accuracy of 98.1% ± 0.7%, while the LRO_DLM-TL model achieved an accuracy of 97.8% ± 1.4%. Table 2 presents the performance outcomes of the LRO_DLM-CE and LRO_DLM-TL models with and without data augmentation. While the LRO_DLM-TL and LRO_DLM-CE models exhibited comparable performance in edge cases, both models demonstrated superior performance to the correlation-based method, particularly when data augmentation was applied.

**Table 2:** Identification accuracy in leave-run-out (LRO) scenarios.

Method	Accuracy
Baseline	0.793
LRO_DLM-CE	0.903 ± 0.018
LRO_DLM-TL	0.917 ± 0.036
LRO_DLM-CE (augmented 90 s)	0.981 ± 0.007
LRO_DLM-TL (augmented 90 s)	0.978 ± 0.014

### 3.3 Performance benchmarking for LSO splitting

In the LSO scenario, the LSO_DLM-TL model demonstrated significant generalization capabilities. Using a fixed 270-s data augmentation segment size and a six-fold cross-validation protocol, the analysis was repeated 10 times, which is consistent with the LRO approach. The model achieved an accuracy of 99.7 ± 0.5. It is worth noting that this high accuracy was achieved using a steep split ratio of 1/6, with 23 subjects in the test set and 138 in the training set. To provide a comprehensive overview of the mode's performance under those optimal conditions, key metrics are summarized in Table 3.

**Table 3:** Performance metrics for DLM-TL in LSO splitting with six-fold cross-validation and data augmentation (270 s).

Method	Accuracy	Precision	Recall	F1 - score	N-subjects train/test-
LSO_DLM-TL (augmented 270 s)	0.997 ± 0.005	0.998 ± 0.004	0.997 ± 0.005	0.997 ± 0.005	115/23

**Figure 3:**
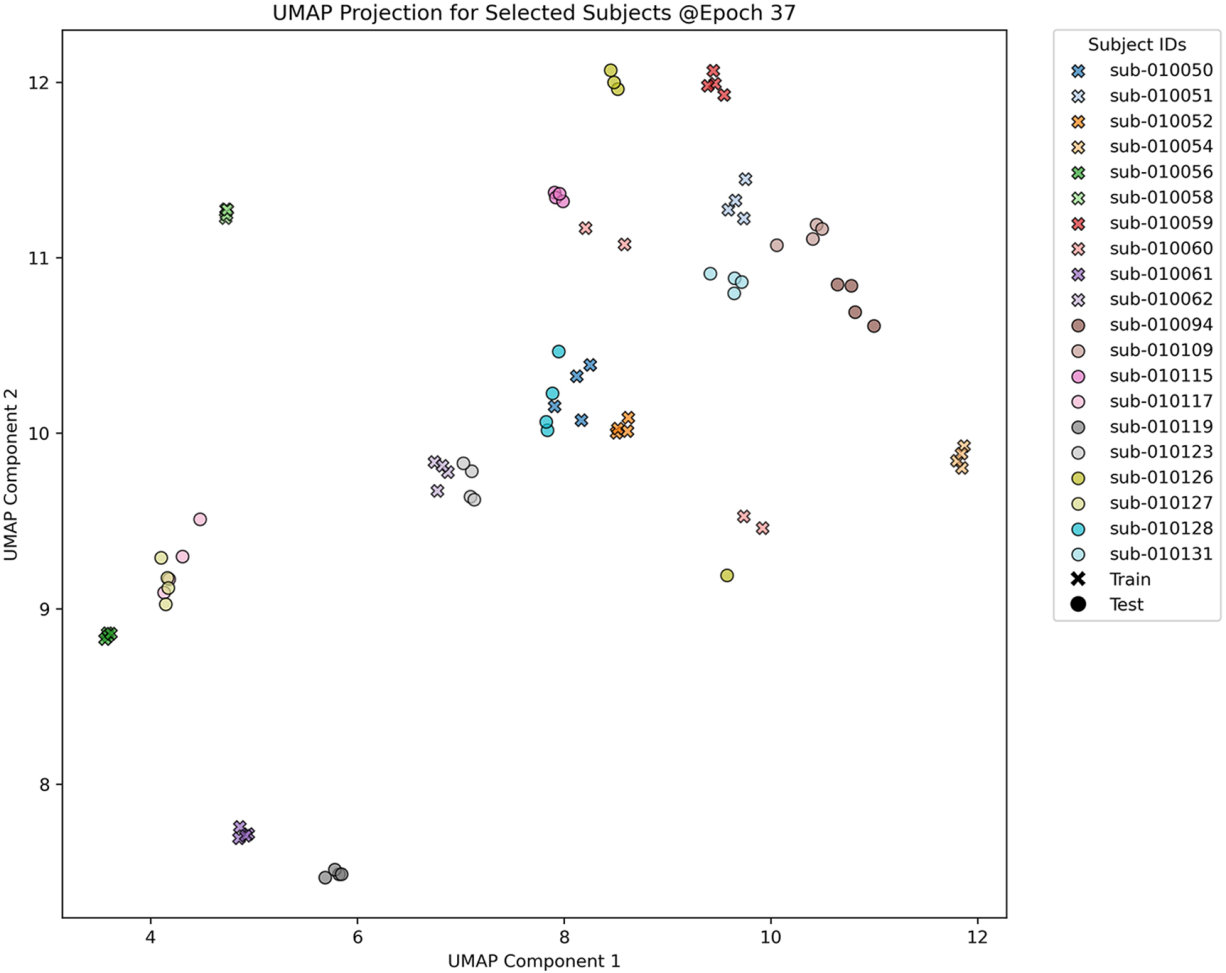
UMAP Visualization of the 256-dimensional embeddings generated by the LSO_DLM_TL model, trained on 270-s data segments over a total of 50 epochs. The plot illustrates a two-dimensional projection at epoch 37, chosen for its lowest validation loss. Each point on the plot represents data from an individual subject. Training and test set data are indicated by crosses and circles, respectively, while different colors correspond to different subject IDs, facilitating the observation of clustering patterns. To enhance visual clarity, only 10 exemplary subjects from each set are displayed. A more comprehensive analysis, including the evolution of all 137 subjects over multiple epochs, is provided in the Supplementary Material.

As illustrated in Figure 3, the UMAP visualization provides a two-dimensional representation of the 256-dimensional embeddings obtained from the LSO_DLM_TL model using 270-s data segments. In this visualization, training data is denoted by crosses, and test data by circles, with different colors representing different subject IDs. The visualization illustrates a discernible clustering of subjects, indicating that the mode's embeddings can effectively capture subject-specific features. It is noteworthy that training and test subjects from the same class tend to cluster closely, demonstrating the mode's ability to generalize well across both sets. For clarity, only 10 exemplary subjects from each set are shown.

#### 3.3.1 Influence of the split ratio

**Figure 4:**
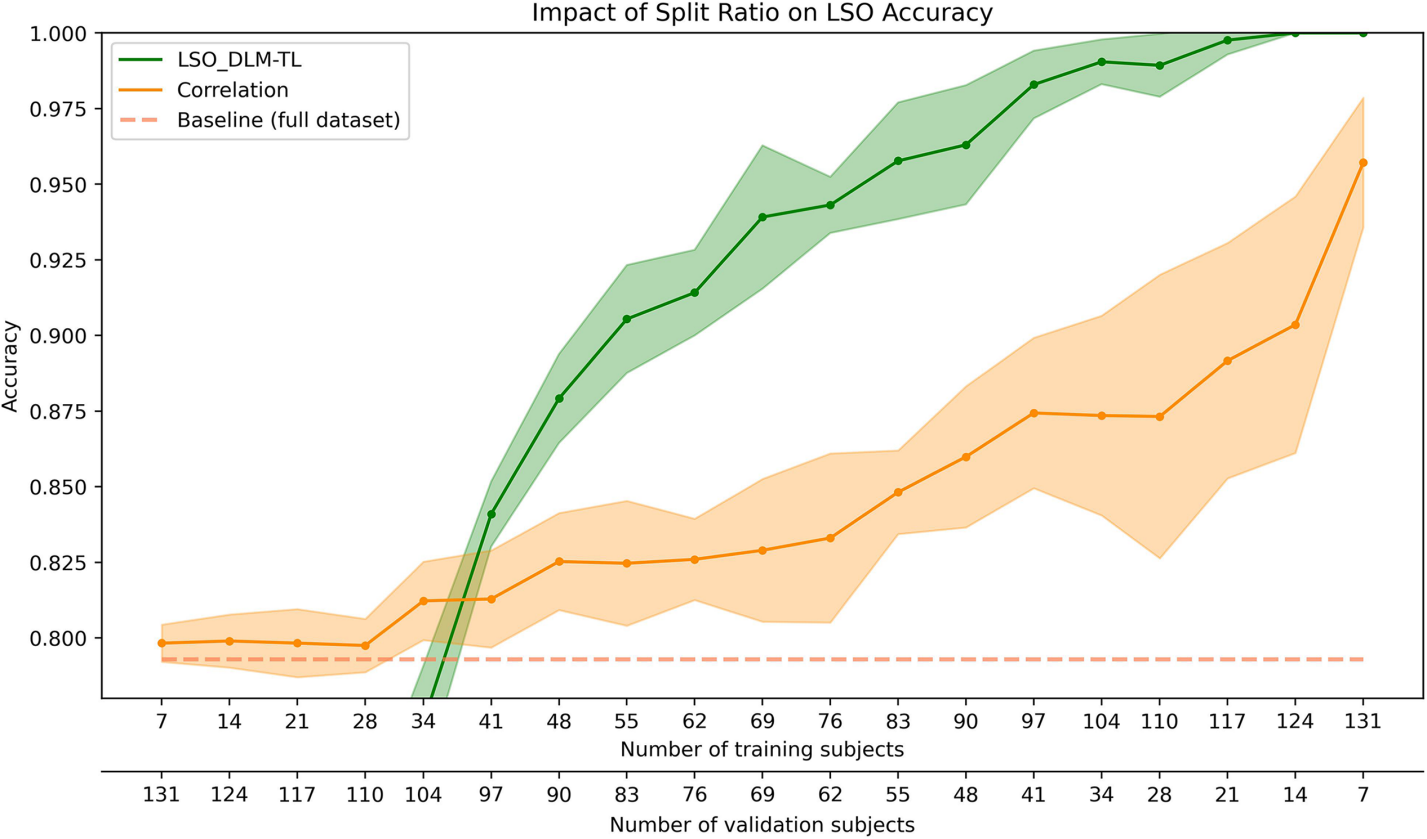
Impact on the identification accuracy with varying split ratios for the training and validation set (indicated by the two x-axes). The performance of the LSO_DLM-TL model (green) is compared against the correlation-based identification (orange). The accuracy is shown for the validation set, except for the baseline (dashed line), which uses Perso's correlation on the full data set. The shaded regions represent the standard deviation for each method.

The relationship between classification performance and different sample sizes in the training and validation sets is illustrated in Figure 4. As the number of training subjects increases, there is a noticeable improvement in model accuracy, reaching a plateau near the size of the full dataset. A decrease in standard deviation with larger training sizes indicates increased model stability. The deep learning approach outperforms the correlation-based baseline when at least 41 subjects, or approximately 30% of the dataset, are used for training. Two primary observations can be made from this data. First, a larger training dataset can improve accuracy and give a more consistent performance across different dataset splits. Second, the increase in correlation performance may be due to the reduced number of subjects in the validation set, while the improvement in model performance is influenced by both the reduction in validation subjects and the addition of more training data.

#### 3.3.2 Impact of dataset size

**Figure 5:**
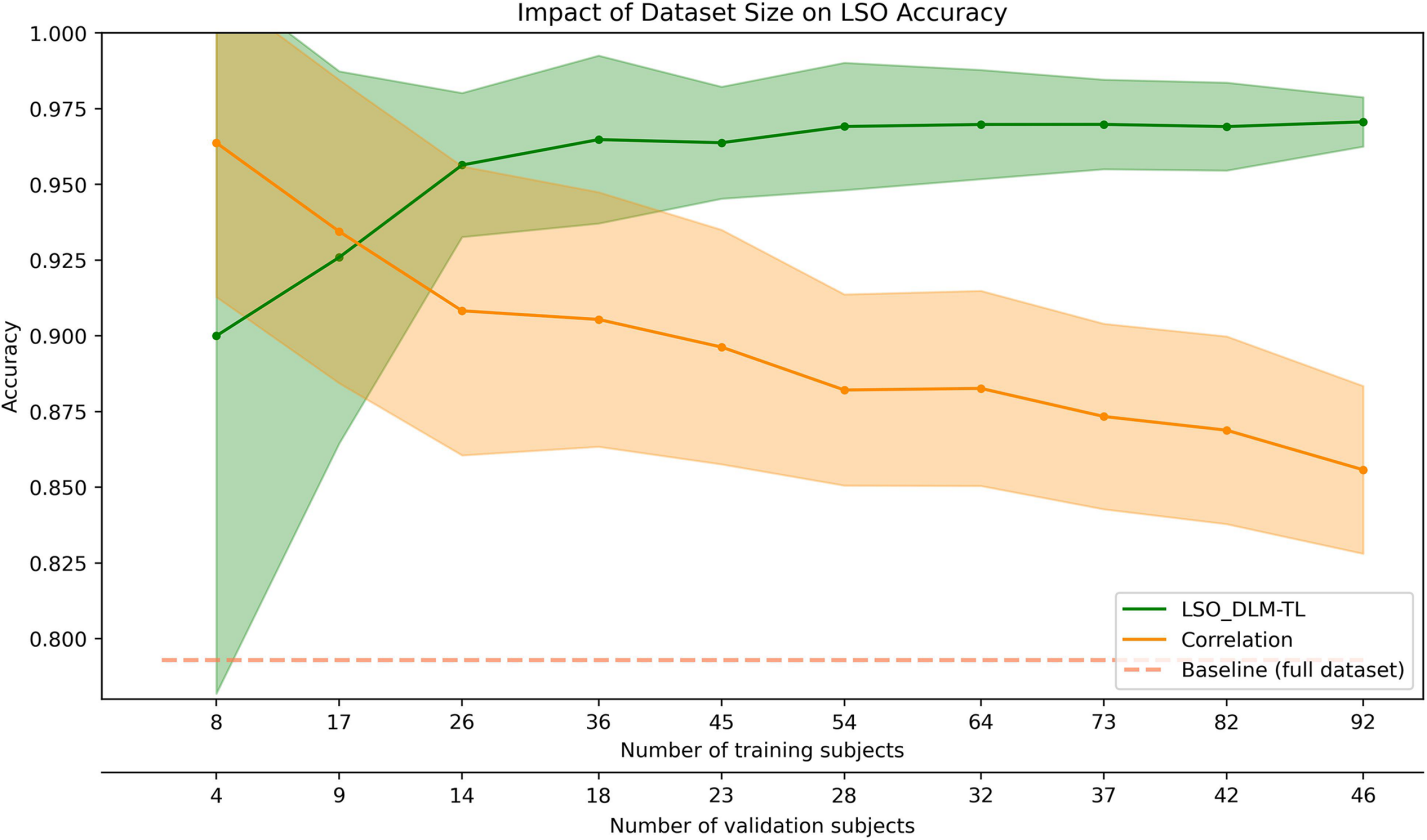
The performance of the LSO_DLM-TL model (green) is compared against the correlation-based identification (orange). The accuracy is shown for the validation set, except for the baseline (dashed line), which uses Perso's correlation on the full data set. The shaded areas represent the standard deviation for each method.

Figure 5 shows that the LSO-DLM-TL model demonstrates superior performance compared to the correlation-based approach when the dataset size reaches 40 subjects. As the number of subjects increases, the accuracy of the model stabilizes, achieving a mean of 97.1% ± 0.8% at 138 subjects (46 of which were used for validation). Convergence is observed around 82 subjects, accompanied by a reduction in the standard deviation. The model exhibits its least optimal performance at 90% ± 11.8% with 12 subjects (4 used for validation).

The figure also illustrates that the correlation-based approach demonstrates optimal performance on very small datasets, achieving an accuracy of 96.4% ± 5.1% with four validation subjects. However, its performance declines monotonically as the dataset size increases, with its poorest performance at 85.6% ± 2.8% when 46 subjects are used for validation. The LSO-DLM-TL model demonstrates a consistent performance advantage over the correlation approach for datasets of 40 subjects or more, while the correlation approach exhibits a decline in performance as the dataset grows.

## 4 Discussion

The primary objective of this study was to address key challenges in neural fingerprinting, particularly the necessity for model retraining and the constraints posed by small datasets. By employing a contrastive learning approach and implementing a data augmentation technique to introduce more variability to the model, we demonstrated the potential of deep learning models to significantly improve subject identification accuracy, even under limited data conditions. Our findings indicate that the LSO_DLM-TL model not only enhances performance but also generalizes well across different subjects without the need for extensive retraining. The contrastive triplet loss model demonstrated robust performance in the leave-subject-out scenario, aligning well with the flexibility of correlation-based methods by offering adaptability to new subjects. These advancements underscore the feasibility of using deep learning for neural fingerprinting in practical settings, where data availability is often limited.

As a reference for the identification performance, we utilized the commonly employed method of correlation analysis as a baseline for classifying subjects according to their functional connectivity profiles. Although the correlation-based accuracy is relatively low compared to previous publications (6, 8, 9), ranging from 93% to 98%, it must be interpreted in relation to the number of subjects and the number of parcellations used. According to Li et al. (9), these two variables have opposite influences on classification accuracy: an increased number of parcellations potentially increases accuracy, while a larger sample size tends to decrease accuracy.

We further investigated the identification performance using a classical multi-class deep learning-based classification paradigm. To facilitate comparison with other studies, the network architecture was adapted from the current state-of-the-art fingerprinting classification on FC profiles using resting-state functional magnetic resonance imaging (rs-fMRI). In this model, a feed-forward network is employed (10). For the dataset we used in this study, this architecture using binary cross-entropy loss achieved a mean accuracy of 90.3 ± 1.8%. This approach, as anticipated, markedly enhanced the baseline performance by approximately 11%. However, the results did not achieve the same level of performance as those reported in other models discussed in the current literature (10, 12), especially without the use of data augmentation. Even after adjusting the hyperparameters, we were unable to achieve the same level of accuracy as compared to previous studies (10, 12) on the LEMON dataset using only 39 parcellations. A possible explanation for this may be due to the fact that the number of parcellations in previous studies was higher compared to our study (Table 4). This supports that both the choice of parcellation and the number of subjects is crucial to performance.

**Table 4:** Comparative analysis of fingerprinting accuracy between augmented LRO_DLM-CE and LRO_DLM-TL models and prior deep learning approaches (1, 7, 12).

Study	DL model	Accuracy	Dataset	*N* subjects	Parcellation size
Ours	LRO_DLM-CE	0.981 ± 0.007	LEMON	138	39
	LRO_DLM-TL	0.978 ± 0.014	LEMON	138	39
(12)	ConvRNN	0.985	HCP	100	236
(10)	corrNN normNN	0.996–0.998	HCP	100	379

While Li et al. (9) explored the impact of subject number and parcellation on fingerprinting, and Sarar et al. (10) looked into the influence of time points and parcellation on feed-forward models for fingerprinting, we extend their work by demonstrating the effectiveness of augmenting temporal information to compensate for limited spatial information. We applied data augmentation by cropping time segments and observed a significant increase in accuracy without the need to increase the partition size. It was found that the fingerprinting accuracy with the LRO_DLM-CE was highest when using FC derived from the shortest time segment of 90 s. Reducing the segment size further resulted in negligible changes in accuracy, based on our empirical observations. However, this finding suggests that considering treating time segment size as a hyperparameter for the DL model could improve the results.

Furthermore, we reformulated the neural fingerprint classification task as a similarity learning task by applying a contrastive learning paradigm (see Section 2.6.2). The DLM-TL models utilize a triplet of FC profiles, comprising an anchor, a positive, and a negative data sample, to assess similarities (i.e., classification) among the FC profiles. A comparison of the performance of contrastive learning (i.e., LRO_DLM-TL) with the classification performance using cross-entropy loss (LRO_DLM-CE) with and without maximally augmented data, revealed that both models yielded comparable results when tested under the usual LRO splitting scenario. Notably, the parameterization with triplet loss (LRO_DLM_TL) demonstrated a substantial improvement in performance, even when only relatively minor augmentation was applied (Figure 2). Nevertheless, contrastive learning is particularly advantageous for LSO re-sampling and allows for learning generalizability to classify unseen classes (i.e., subjects). In contrast to the widely recognized acknowledgment that deep learning-based models perform better with larger data, the contrastive learning paradigm with LSO sampling presented comparable performance to the state-of-the-art (10) with a much smaller parcellation size and a moderately larger set of subjects.

### 4.1 Influence of dataset size

In contrast with the prevailing assumption that extensive datasets are necessary for the optimal functioning of deep learning models, our experimental setup (comprising 39 ROIs and 15 min of resting-state data) has yielded notable enhancements in model-based fingerprinting relative to conventional correlation-based techniques, with datasets comprising a minimum of about 80 subjects. We attribute this outcome to the intrinsic stability of the fingerprinting process. Two opposing factors influence the accuracy of the model with respect to dataset reduction. First, as the number of subjects in the training set decreases, the mode's ability to learn inter-individual differences from the training data and generalize these differences to new individuals is compromised. Conversely, this decline in performance is compensated by the associated simplification of the multi-class classification problem when the number of subjects in the validation set decreases at a similar rate (Figure 5). This latter effect mirrors its counterpart in correlation-based methods and is consistent with the observations of Li and colleagues who found that a decrease in the number of subjects led to less cluttering of functional connectivity in a high-dimensional space (9). This robustness may prove advantageous in scenarios where collecting large amounts of data is either impractical or cost prohibitive.

When evaluating the accuracy of fingerprinting in the LSO context, both of these factors deserve attention due to their combined effect on the sensitivity of the model to different split ratios. Our analysis reveals that commonly used split ratios in the range of 2/3–3/4 offer significant accuracy improvements in comparison to the correlation-based approach (Figure 4). In addition, accurate determination of the most appropriate split ratios can improve the effectiveness of model training, allowing researchers to make full use of existing data.

### 4.2 Potential applications

The integration of a contrastive learning framework enhances the flexibility of deep learning models for the LSO-split use case. Through a single training session, the model can effectively acquire the ability to condense raw connectivity data into reusable embeddings, which may be considered as fingerprints. While the current study utilized these embeddings solely for identification, future investigations could explore their potential to capture diverse subject characteristics and function as potential biomarkers. Although the present study employed these embeddings exclusively for identification purposes, future research could investigate whether they can capture various characteristics of a subject and serve as potential biomarkers. If validated, training identity as a pretext task within a large-scale transfer learning context would present an appealing opportunity. This approach would leverage a significant amount of publicly available data, containing subject identifiers in their original form, for the purpose of semi-supervised training. A potential clinical application might involve utilizing consecutive annual functional measurements to monitor consistency (self-similarity) and identify early signs of disease or cognitive decline.

The study investigates a generic augmentation method that demonstrates adaptability and potential extension to other neuroimaging modalities. This approach effectively addresses the trade-off between spatial and temporal resolutions, enhancing model performance to meet specific needs. The flexibility of this approach enables the model to analyze a wide range of spatio-temporal data types at various levels. This versatility is essential for models to generalize effectively across diverse and smaller datasets, facilitating their application in various research settings.

The integration of contrastive learning and data augmentation techniques has been shown to improve the adaptability and generalization capabilities of deep learning models in neuroimaging. This has significant potential for applications across a wide range of modalities.

### 4.3 Limitations and future directions

The principal limitation of this study (*n* = 138) is its generalizability. In the context of this study, the term “generalizability” refers to the mode's ability to distinguish between subjects who were not included in the training dataset with high accuracy. It is important to clarify that this definition does not encompass the mode's capability to generalize to data from various sites or sources. Furthermore, the longevity of neural fingerprints was not evaluated over extended periods, as the dataset lacked longitudinal scans. This presents a significant limitation, as exploring inter-subject variability over time would provide insights into the long-term stability of neural fingerprints and their reliability in fingerprinting tasks.

A crucial consideration is the relationship between data size and model performance. It is commonly suggested that deep learning models exhibit improved performance when exposed to larger datasets. However, it is important to differentiate between wide data, which refers to a greater number of recordings, and large data, which includes a larger number of subjects. This differentiation is especially pertinent when assessing the accuracy of a model in the fingerprinting task, particularly when introducing new subjects. Our findings indicate that as the complexity of the classification problem increases, the challenge of achieving high performance in neural fingerprinting tasks also escalates. In conclusion, while our model can differentiate between unseen subjects, its ability to generalize to other datasets or sites remains untested and is potentially limited. Future research should prioritize on evaluating model performance across diverse datasets and sources to enhance our comprehension of its generalizability across datasets and potential applications in broader contexts.

## 5 Conclusion

In conclusion, we have successfully employed contrastive learning and data augmentation techniques in a deep learning-based data analysis to overcome limitations caused by a limited data pool, resulting in robust identification performance. Specifically, we focused on its utility in multi-class classification scenarios, such as neural fingerprinting, as well as in data analysis settings with small sample sizes or specific re-sampling scenarios (LRO, LSO). Through data augmentation and a contrastive learning technique, we have achieved an accuracy of about 98% in identifying a single subject out of 138 subjects from 39 different functional connectivity profiles and have demonstrated the potential of the method for generalization to unseen subjects or data. This approach could, for example, be used in the future to estimate the similarity of data from a new subject (unknown to the model) to data representing the control (normal data) or experimental (e.g., abnormal data) group. The measure of similarity is, in principle, adaptable and flexible across different data types and neuroimaging modalities.

## Data Availability

Publicly available datasets were analyzed in this study. This data can be found here: https://doi.org/10.1038/sdata.2018.308.

## Supplementary material

Supplementary Figure S1 UMAP projections of the 256-dimensional embeddings generated by the LSO_DLM_TL model for selected subjects across three epochs (3, 15, and 37) are presented. Each plot illustrates a two-dimensional projection of the embeddings derived from 90-second rs-fMRI data segments. Individual points represent data from subjects, with training set data indicated by crosses and test set data indicated by circles. Different colors correspond to different subject IDs, facilitating the visualization of clustering patterns for each subject. The evolution of the clustering is depicted from the early training stages (epoch 3) through intermediate training (epoch 15) to near completion (epoch 37), highlighting how the model's improving ability to differentiate between subjects over time.
