# Supplementary figures and images for: Contrastive learning for neural fingerprinting from limited neuroimaging data
Source: Front Nucl Med. 2024 Nov 13;4:1332747. doi: 10.3389/fnume.2024.1332747 (PMC11598699; doi:10.3389/fnume.2024.1332747)

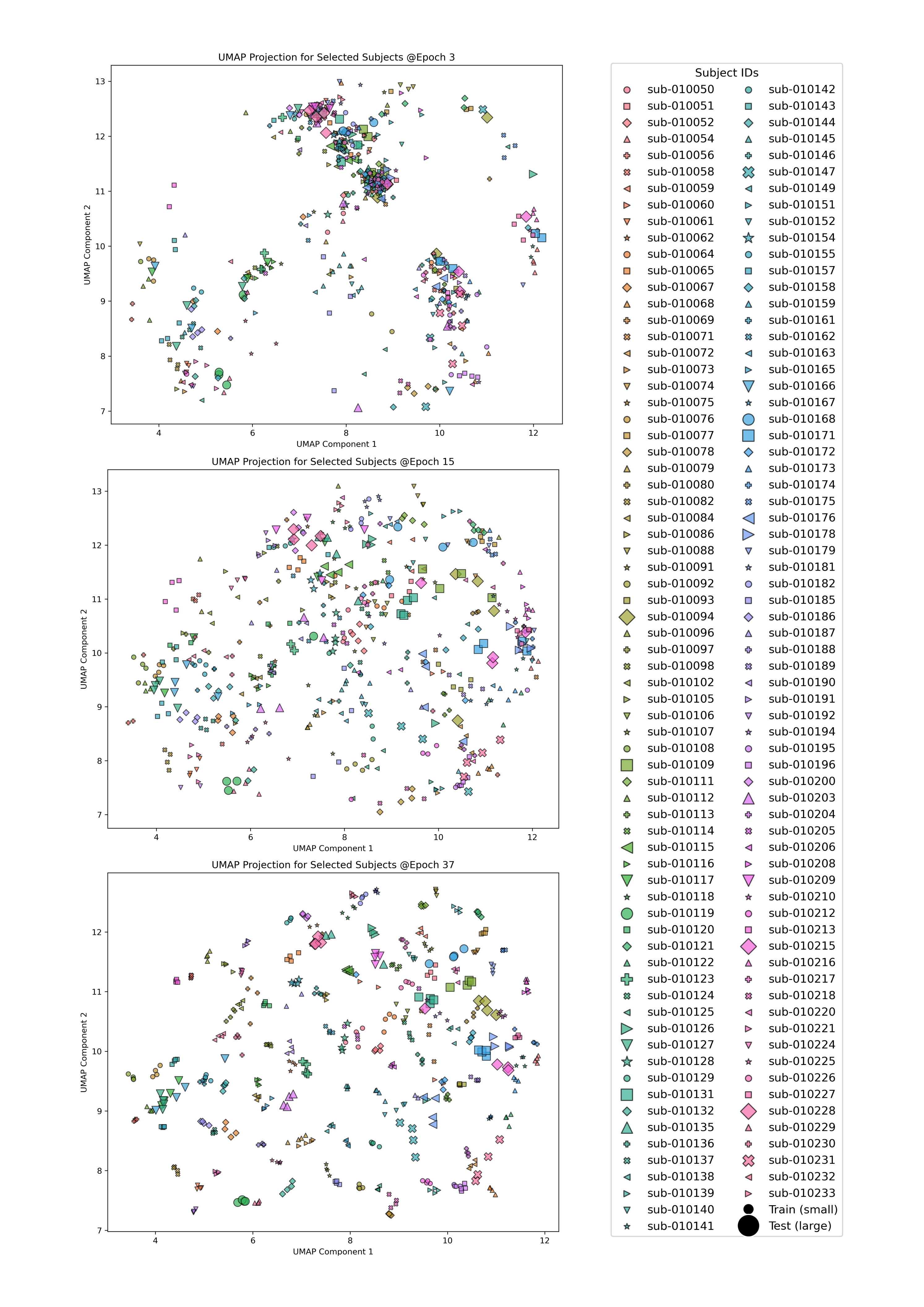

Supplement: Supplementary Figure S1 — UMAP projections of the 256-dimensional embeddings generated by the LSO_DLM_TL model for selected subjects across three epochs (3, 15, and 37) are presented. Each plot illustrates a two-dimensional projection of the embeddings derived from 90-second rs-fMRI data segments. Individual points represent data from subjects, with training set data indicated by crosses and test set data indicated by circles. Different colors correspond to different subject IDs, facilitating the visualization of clustering patterns for each subject. The evolution of the clustering is depicted from the early training stages (epoch 3) through intermediate training (epoch 15) to near completion (epoch 37), highlighting how the model's improving ability to differentiate between subjects over time. [file Image1.jpeg]
